# Laboratory evaluation of a prospective remediation method for PCB-contaminated paint

**DOI:** 10.1186/2052-336X-12-57

**Published:** 2014-03-06

**Authors:** Erin K H Saitta, Michael J Gittings, Christian Clausen, Jacqueline Quinn, Cherie L Yestrebsky

**Affiliations:** 1 Department of Chemistry, University of Central Florida, 4000 Central Florida Blvd, Orlando, FL 32816-2366, USA; 2 John Jay College of Criminal Justice, 899 Tenth Avenue, New York, NY 10019, USA; 3 National Aeronautics and Space Administration, Kennedy Space Center, Florida 32899, USA

**Keywords:** Polychlorinated biphenyl (PCB), Remediation, Aroclor, Paint, Magnesium

## Abstract

**Background:**

Paint laden with polychlorinated biphenyls (PCBs) often acts as a point source for environmental contamination. It is advantageous to address contaminated paint before the PCBs transport to surrounding media; however, current disposal methods of painted material introduce a variety of complications. Previous work demonstrates that PCBs can be broken down at ambient temperatures and pressures through a degradation process involving magnesium metal and acidified ethanol. This report is an extension of that work by describing the development of a delivery system for said reaction in preparation for a field test. Two treatment options including the Activated Metal Treatment System (AMTS) and the Non-Metal Treatment System (NMTS) remove and degrade PCBs from painted surfaces.

**Findings:**

AMTS decreased the Aroclor® concentration of a solution by more than 97% within 120 minutes and the Aroclor® concentration of industrial paint chips by up to 98% over three weeks. After removing up to 76% of PCBs on a painted surface after seven days, NMTS also removed trace amounts of PCBs in the paint’s concrete substrate. The evaporation rate of the solvent (ethanol) from the treatment system was reduced when the application area was increased. The solvent system’s ability to remove more than 90% of PCBs was maintained after losing 36% of its mass to solvent evaporation.

**Conclusions:**

The delivery systems, AMTS and NMTS, are able to support the hydrodechlorination reaction necessary for PCB degradation and are therefore attractive options for further studies regarding the remediation of contaminated painted surfaces.

## Findings

### Introduction

Despite regulation, PCB-contaminated materials remain prevalent in the environment including remote locations [1,2]. Renovations and weather conditions cause PCB-contaminated paint to flake, leading to increased concentrations in water and soil [3-5]. Once in the soil, addressing PCBs through dredging, capping and bioremediation is difficult and expensive therefore removing PCBs from painted surfaces before they enter the environment is advantageous [6]. Unless under the EPA action limit, federal law requires PCB-contaminated materials be disposed of in a limited number of licensed landfills, where they often charge by amount of material disposed [7].

Many PCB-contaminated sites contain large structures with painted vertical surfaces. Remediating these sites often require the structures to be broken down and transported, an expensive process that can further contaminate the environment. Incineration of the PCB-contaminated materials can emit other toxic compounds like dibenzodioxins and dibenzofurans [8]. The PCB-contaminated dust that is produced by sandblasting has been reported to spread to surrounding surfaces and the environment [9]. Considering the issues introduced by current remediation techniques, a novel remediation option for painted structures is desired.

Previous research has shown that magnesium, carboxylic acid, and alcohol have the ability to degrade polycyclic aromatic hydrocarbons, like PCBs, through hydrodechlorination [10-12]. This report describes an extension of that research with the development of a delivery system for the reaction components creating degradation options for PCB-contaminated painted structures. The Activated Metal Treatment System (AMTS) and a Non-Metal Treatment System (NMTS) are formulated to be applied to painted surfaces and sealed to minimize evaporation [13]. Calcium stearate, polyethylene glycol, glycerol, and sodium polyacrylate were added to ethanol, limonene and acetic acid to create an application media viscous enough to adhere to a vertical surface. Magnesium metal in the AMTS begins degrading PCBs as they enter the system while the NMTS extracts PCBs from a surface and is subsequently combined with metal to degrade PCBs.

### Experimental

#### Aroclor® degradation through AMTS

**Figure 1:**
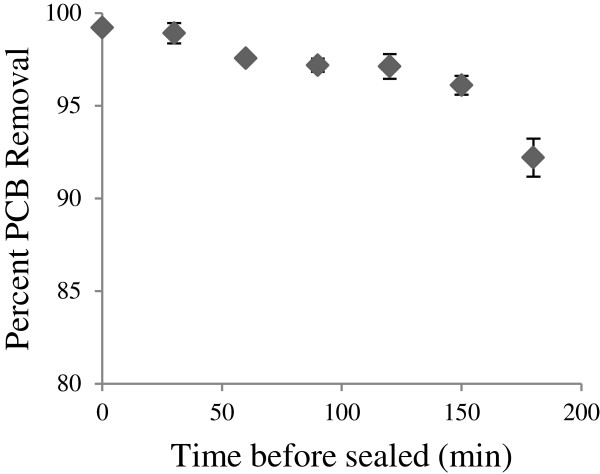
**Concentration of Aroclor** ***® *** **1260 over time during exposure to Activated Metal Treatment System (AMTS).**

A 10.0 μl aliquot of 12,500 ng/μl Aroclor® 1260 was added to individual vials containing 0.50 g of AMTS. After the time displayed in Figure 1, samples were extracted and analyzed.

#### Aroclor® degradation in contaminated paint chips

A 0.8 g aliquot of AMTS was added to 0.15 g of industrial paint chips contaminated with a mixture of Aroclor® 1248 and 1260. After the time displayed in Table 1, the mixture of AMTS and paint chips were extracted and analyzed.

**Table 1:** PCB concentrations of industrial paint chip samples reacting with AMTS for 1 and 3 weeks

**Sample location #**	**Initial concentration (mg/Kg)**	**7 Day concentration (mg/Kg)**	**3 Week concentration (mg/Kg)**
1	1390 ± 152	392 ± 31.0	42.6 ± 23.5
2	831 ± 42.0	433 ± 136	51.2 ± 15.5
3	2290 ± 132	491 ± 155	104 ± 21.0
4	2397 ± 16.2		52.3 ± 5.77
5	2780 ± 88.5		55.7 ± 9.01
6	4540 ± 181		385 ± 43.0
7	1000 ± 42.0		21.6 ± 0.30
8	1380 ± 79.0		42.0 ± 28.8

#### Remediation of painted surface

Approximately 100 mg of Aroclor® 1254 was added to 0.182 kg of Olympic® fast hide with ultra semi-gloss paint. Three coats of paint were applied in 3.5 cm^2^ sections to two concrete blocks with a 24-hour drying period in between each coat. One block was treated with the NMTS and sealed while the other was left untreated as a control. Both blocks were sampled in triplicate after three and seven days. A 12.7 mm masonry drill bit was used to sample the concrete at two depths, 0 mm-8 mm and 8 mm-18 mm respectively. The paint and concrete were extracted and analyzed for PCBs.

#### PCB removal based on solvent evaporation

**Figure 2:**
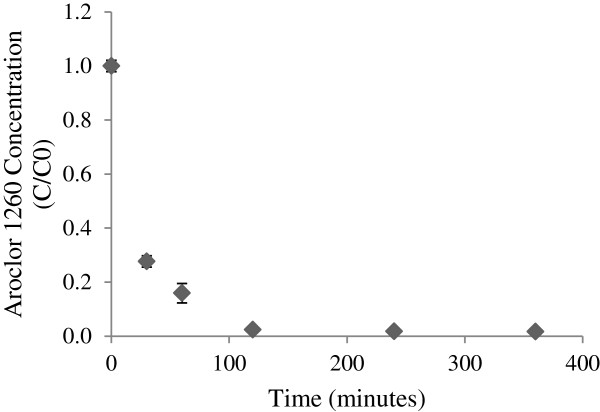
Percent PCB removal from paint relative to the amount of time passed before sealing the treatment system.

PCB-laden paint, made by combining 15 mg of PCB congener 151 with 0.073 kg of Olympic® paint, was applied to an aluminum surface in 2 cm^2^ areas and allowed to dry. A 3 cm^2^ area of NMTS was applied to each paint sample and was sealed with a vinyl polymer in thirty minute intervals. The amount of time that the NMTS remained exposed to ambient conditions before being sealed is displayed in Figure 2. The painted aluminum surfaces were extracted and analyzed for PCBs three days after the initial application.

#### PCB extraction and analysis

Samples were extracted in 10.00 ml of toluene using ultrasonic extraction [14]. Samples were centrifuged and the supernatants were subjected to a sulfuric acid/permanganate clean-up [15]. Analysis was done in duplicate, unless stated otherwise, utilizing a Perkin Elmer AutoSystem XL GC/FID/ECD outfitted with a 30 m Restek Rtx-5 column (0.25 mm ID, 0.25 um df). The temperature was ramped from 120°C to 300°C. Aroclor® concentrations were quantified by summing the area of five characteristic peaks in the mixture [16].

### Results & discussion

#### Aroclor® degradation through AMTS

Figure 1 shows the degradation that occurred within the delivery system containing the hydrodechlorination reaction components, bulking agents, and paint softener. The AMTS degraded the majority of the peaks used for Aroclor® 1260 quantification within 120 minutes supporting the claim that the treatment technology is a suitable matrix for significant PCB degradation. This timeframe is optimistic considering Single congener PCB-151 (2,2′,3,5,5′,6-PCB) has been reported in literature to degrade in the simpler matrix of ethanol, acetic acid, and magnesium within an hour [11]. The degradation is fast considering other popular techniques, like bioremediation, can take over 120 days to dechlorinate higher chlorinated PCB congeners by only 67% [17,18]. Description of components and preparation of the treatment system are described in Additional file 1 and Additional file 2: Figure S1.

#### Aroclor® degradation in contaminated paint chips

Analyzing paint samples is often challenging as great variations in PCB concentrations can occur, even within small test areas, due to the weathering of the paint, past renovations, and inconsistent paint applications [4]. The degradation of PCBs in paint chips can be seen in Table 1. Paint chips were sampled from structural materials and machine parts at eight locations of an aged manufacturing facility. Three samples were extracted after seven days of exposure to AMTS at which point approximately 21–52% of the original PCB concentration remained. Samples from all of the locations were extracted after three weeks of exposure to AMTS at which point approximately 2-8% of the original PCB concentration remained. Other degradation techniques reported to have been used on Aroclor contaminated paint require extreme conditions like high temperatures and pressures [19].

#### Remediation of painted surface

**Figure 3:**
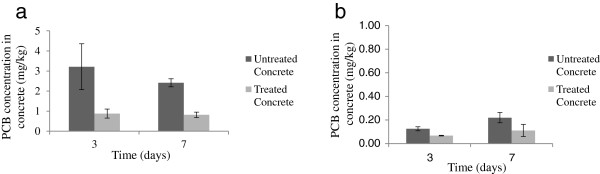
**PCB concentration in concrete over time at various depths. (a)** Concrete 0mm-8mm below the surface **(b)** Concrete 8mm-18mm below the surface.

An analysis of the treatment system’s ability to remove PCBs from painted porous material was conducted on concrete. After three days of treatment, approximately 73% of the PCBs in the paint were removed by the NMTS which rose to 76% removal at the end of day seven. Regarding the penetration into the concrete, Figure 3a and 3b show that at three and seven days, fewer PCBs were detected in the treated concrete at both depths tested. As opposed to the PCBs being pushed further into the material during remediation, the NMTS removed PCBs that were there as a result of the original painting process. During the sampling, an effort was made to ensure that all paint was removed from the surface before the concrete was sampled. However, the variation in PCB concentrations for the untreated concrete at a depth of 0–8 mm on day three may be due to a flake of paint contaminating the sample. This small scale experiment simulates a field study where PCBs leached into concrete from nearby contaminated building material. The remediation technique described in the literature involved grinding down the top, and most concentrated, layer of the concrete to remove the contaminant [20]. Although both techniques enable the building to remain intact, our study demonstrates that NMTS can remove the leached PCBs during the treatment of the point source while avoiding further contamination to the surrounding environment.

#### PCB removal based on solvent evaporation

Treating large structures with AMTS/NMTS may require an extended time period between the application of the treatment system and the application of the sealant. The impact of a prolonged delay between the treatment application and the sealing process regarding PCB removal capability was analyzed. Figure 2 displays the percentage of PCB removal relative to the amount of time samples were exposed to ambient conditions before being sealed. It demonstrates that after three hours of exposure to ambient conditions, resulting in a 36% loss of solvent mass described in the Additional file 3 and Additional file 4: Figure S2, the NMTS maintained the ability to remove over 90% of the PCBs in the paint which is comparable to the reported extraction efficiencies of PCBs in soil [21]. Therefore, on a large scale, the time it would take to apply the treatment system to an entire wall/structure and the time it would take to seal the system would not greatly inhibit PCB removal.

### Conclusion

The analysis of the treatment systems attests to the fact that the Activated Metal Treatment System (AMTS) and Non Metal Treatment System (NMTS) are feasible remediation options. Aroclor® mixtures in solution and paint chips displayed significant degradation in the treatment system when activated with magnesium and acidified ethanol. Applied to painted porous materials, the treatment system decreased trace amounts of PCBs below the painted surface. A vinyl polymer sealant was used to minimize solvent loss and encourage PCB removal. Even when the time between the application and the sealing process was extended (simulating the treatment needs of large structures) high treatment efficiency was achieved. The information provided could eliminate the need to demolish and transport contaminated structures by providing a quick cost effective remediation technology. Once under the regulatory action limit, treated materials would be able to be repainted, used again, or resold.

## Abbreviations

(PCBs): Polychlorinated biphenyls; (AMTS): Activated metal treatment system; (NMTS): Non-metal treatment system; (EPA): Environmental Protection Agency; (GC): Gas chromatograph; (FID): Flame ionization detector; (ECD): Electron capture detector.

## Competing interests

The United States of America as represented by the Administrator of the National Aeronautics and Space Administration holds the rights to the patent. CC, JQ, and CY are listed as co-inventors on the patent.

JQ is a NASA employee but as a government employee, does not receive remuneration for intellectual property discoveries. The other authors do not receive reimbursements, fees, funding, or salary or any other financial benefit from this intellectual property.

The authors declare that they have no competing interests.

## Authors’ contributions

CC, JQ, and CY assisted in the conception/design of the technology and edited the manuscript. CY participated in the experimental design, the analysis of results, and the drafting of the manuscript. ES lead the laboratory studies, sample extraction and analysis of results as well as the drafting, writing and revising of the manuscript. MG assisted in the experimentation portion of the laboratory studies, sample extraction, analysis of results and editing of the manuscript. All authors read and approved the final manuscript.

## Supplementary Material

Additional file 1 **Treatment system components and preparation.** This information describes how to prepare the treatment system described in the report. It is accompanied by Additional file 2: Figure S1. Click here for file

Additional file 2: Figure S1 Schematic diagram to describe the production NMTS and AMTS. Click here for file

Additional file 3 **Evaporation rate as a function of surface area.** This information describes and analyses data that observed the evaporation rate of the solvent system as a function of surface area of the treatment system. It is accompanied by Additional file 4: Figure S2. Click here for file

Additional file 4: Figure S2 Percent solvent loss over time for samples with a surface area to mass ratio of ∆3.6 cm^2^/g, ∆1.8 cm^2^/g, and ∆1.3 cm^2^/g. Click here for file
