# Supplementary material for: Laboratory evaluation of a prospective remediation method for PCB-contaminated paint
Source: J Environ Health Sci Eng. 2014 Mar 6;12:57. doi: 10.1186/2052-336X-12-57 (PMC4108127; doi:10.1186/2052-336X-12-57)
Supplement: Additional file 1 — Treatment system components and preparation. This information describes how to prepare the treatment system described in the report. It is accompanied by Additional file 2: Figure S1. [file 2052-336X-12-57-S1.docx]

***Treatment system components***

The components of the application medium were chosen with regard to environmental impact and remediation chemistry. Ethanol was selected as the predominant solvent because it is environmentally safe, solubilizes PCBs and is an effective solvent for the PCB degradation process [11]. Limonene, a natural paint softener, was added to open the polymeric lattice of the paint and enables the PCBs to migrate into the treatment system. The appropriate viscosity was achieved by the addition of bulking agents calcium stearate, polyethylene glycol, and glycerol. Lastly, sodium polyacrylate, a super absorbent polymer, was added to retain the solvent necessary for PCB removal and degradation.

***Treatment system preparations***

When the treatment systems were prepared, a series of three different mixtures were set up in three separate containers before being combined together to produce the final formulation as seen in Additional file 3: Figure S1. Both treatment systems, AMTS and NMTS, were made by combining 68.16 ml of absolute ethanol, 7.58 ml of limonene, and 0.765 ml of glacial acetic acid in container 1. In container 2, 10.0 g of calcium stearate were added to 5.0 g of polyethylene glycol 8000. Container 3 consisted of 10.0 g of sodium polyacrylate with 5.0 g of glycerol. Once all of the components were measured, the solvent system in container 1 was poured into container 2 which was added to container 3 and stirred until uniform. The treatment system was poured into an air tight container and sealed. Within thirty minutes the treatment system formed the consistency of a thick paste that could adhere to a vertical surface. The prepared treatment system was used within a year when kept sealed and was either used “as is” in the form of a NMTS or was combined with magnesium metal and acidified ethanol immediately before use to act as AMTS.

To prepare the AMTS, a 20.00 g portion of previously made NMTS was added to container A which consisted of 1.20 g of magnesium metal coated with 1.20 g of glycerol. In container B, 0.224 ml of acetic acid was combined with 2.016 ml of ethanol. The solvents in container B were added to container A, mixed until uniform, and used immediately as AMTS.
