# Supplementary material for: Laboratory evaluation of a prospective remediation method for PCB-contaminated paint
Source: J Environ Health Sci Eng. 2014 Mar 6;12:57. doi: 10.1186/2052-336X-12-57 (PMC4108127; doi:10.1186/2052-336X-12-57)
Supplement: Additional file 2: Figure S1 — Schematic diagram to describe the production NMTS and AMTS. [file 2052-336X-12-57-S2.doc]

**NMTS**

Container 3

Sodium polyacrylate

Glycerol

Container 2

Calcium stearate

Polyethylene glycol

Container 1

Ethanol

Limonene

Acetic acid

Final Container

(Airtight)

Container B

Ethanol

Acetic acid

Container A

Magnesium

Glycerol

**AMTS**
