# Supplementary material for: Laboratory evaluation of a prospective remediation method for PCB-contaminated paint
Source: J Environ Health Sci Eng. 2014 Mar 6;12:57. doi: 10.1186/2052-336X-12-57 (PMC4108127; doi:10.1186/2052-336X-12-57)
Supplement: Additional file 3 — Evaporation rate as a function of surface area. This information describes and analyses data that observed the evaporation rate of the solvent system as a function of surface area of the treatment system. It is accompanied by Additional file 4: Figure S2. [file 2052-336X-12-57-S3.docx]

***Evaporation rate as a function of surface area***

In order to observe the rate at which the solvent evaporated from the NMTS, three samples of NMTS with various surface areas were analyzed over 12 hours. Three square samples of NMTS, each with a thickness of 1.6 cm, were shaped to provide three different surface areas of 69, 39, and 12 cm^2^ (top and sides exposed). The change in mass due to the evaporation process was measured on a Denver Instrument A160 balance.

Additional file 4: Figure S2 displays the percent solvent loss (m/m) as a function of the surface area to mass ratio. Having the same thickness, the distance that the solvent had to travel from the bottom to the top of the matrix was the same for each sample. The length, however, varied and as a result, the ratio of surface area to mass also varied. As the treatment system was applied in larger areas, the surface area to total mass ratio decreased and resulted in a smaller percent solvent loss. Thus, when keeping a consistent thickness, applying the treatment system to a larger area will reduce the percentage of solvent that evaporates over time. These results establish an optimistic foundation for the eventual large scale application of the technology where entire structures may be treated.
