# Supplementary figures and images for: Laboratory evaluation of a prospective remediation method for PCB-contaminated paint
Source: J Environ Health Sci Eng. 2014 Mar 6;12:57. doi: 10.1186/2052-336X-12-57 (PMC4108127; doi:10.1186/2052-336X-12-57)

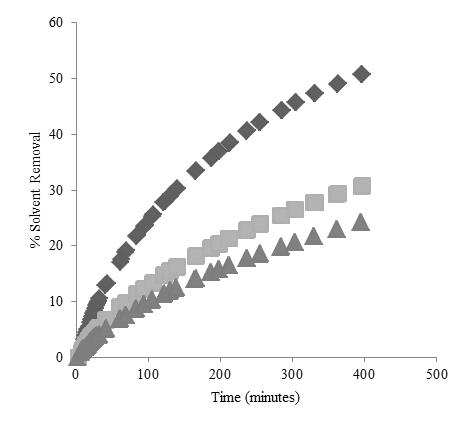

Supplement: Additional file 4: Figure S2 — Percent solvent loss over time for samples with a surface area to mass ratio of ∆3.6 cm2/g, ∆1.8 cm2/g, and ∆1.3 cm2/g. [file 2052-336X-12-57-S4.doc]
